# Highly Sensitive Iontronic Pressure Sensor with Side‐by‐Side Package Based on Alveoli and Arch Structure

**DOI:** 10.1002/advs.202309407

**Published:** 2024-03-15

**Authors:** Zhi Ding, Weijian Li, Weidong Wang, Zhengqian Zhao, Ye Zhu, Baoyin Hou, Lijie Zhu, Ming Chen, Lufeng Che

**Affiliations:** ^1^ College of Information Science and Electronic Engineering Zhejiang University Hangzhou 310027 China; ^2^ Center for Microelectronics Shaoxing Institute Zhejiang University Shaoxing 312035 China

**Keywords:** double‐sided microstructure, graded hollow ball arch, iontronic, side‐by‐side package

## Abstract

Flexible pressure sensors play a significant role in wearable devices and electronic skin. Iontronic pressure sensors with high sensitivity, wide measurement range, and high resolution can meet requirements. Based on the significant deformation characteristics of alveoli to improve compressibility, and the ability of the arch to disperse vertical pressure into horizontal thrust to increase contact area, a graded hollow ball arch (GHBA) microstructure is proposed, greatly improving sensitivity. The fabrication of GHBA ingeniously employs a double‐sided structure. One side uses mold casting to create convex structures, while the other utilizes the evaporation of moisture during the curing process to form concave structures. At the same time, a novel side‐by‐side package structure is proposed, ensuring pressure on flexible substrate is maximally transferred to the GHBA microstructure. Within the range of 0.2 Pa–300 kPa, the iontronic pressure sensor achieves a maximum sensitivity of 10 420.8 kPa^−1^, pressure resolution of 0.1% under the pressure of 100 kPa, and rapid response/recovery time of 40/35 ms. In wearable devices, it is capable of monitoring dumbbell curl exercises and wirelessly correcting sitting positions. In electronic skin, it can non‐contactly detect the location of the wind source and achieve object classification prediction when combined with the CNN model.

## Graphical abstract

Iontronic pressure sensors possess high capacitance characteristics. Based on the alveoli structure to improve compressibility and arch structure to disperse vertical pressure into horizontal thrust, a graded hollow ball arch microstructure is proposed, greatly improving sensitivity to 10 420.8 kPa^‐1^. When combined with circuits and machine learning, the iontronic pressure sensor can wirelessly correct the sitting position and achieve object classification prediction.

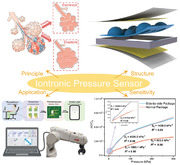

## 1 Introduction

Smart healthcare,^[^ ^1^, ^2^, ^3^, ^4^, ^5^ ^]^ intelligent robotics,^[^ ^6^, ^7^, ^8^, ^9^, ^10^ ^]^ and wearable devices^[^ ^11^, ^12^, ^13^, ^14^, ^15^ ^]^ have all seen rapid progress in recent years, which raised awareness of flexible pressure sensors with tactile sense. In daily life, the bending of joints and the vibration of vocal cords both generate pressure, and the value of pressure can either decrease or increase when our lifestyle habits are unhealthy. For example, when the sitting position is incorrect, the degree of spinal curvature increases, leading to larger pressure; when someone is hoarse, the vibration of the vocal cords weakens, resulting in lower pressure. Flexible pressure sensors as the sensing layer and electronic circuits as the signal processing layer constitute a smart wearable device that can assist people in the real‐time correction of unhealthy lifestyle habits.^[^ ^16^, ^17^ ^]^ Flexible pressure sensors can also serve as the electronic skin for robotic arms. The sensor outputs different resistance or capacitance values when it grasps objects with different hardness and shapes. Machine learning (ML), an important area of artificial intelligence, can process this complex data efficiently. An artificial intelligence (AI) model trained on large amounts of data can serve as the brain of a robot, enabling it to recognize various objects.^[^ ^6^, ^18^ ^]^

Capacitive flexible pressure sensors have the advantages of low power consumption and strong anti‐interference capabilities.^[^ ^19^ ^]^ In recent years, Professor Tingrui Pan from the University of California, Davis, discovered a novel iontronic capacitor composed of chemical ionics and conductor electrons, which offers potential for new applications in flexible touch perception.^[^ ^20^, ^21^ ^]^ The normal parallel‐plate capacitor primarily relies on the variation in distance between the upper and lower electrode plates to affect the capacitance. In contrast, the iontronic capacitor only generates interface capacitance when the ionic gel and the electrode plate come into contact. As a result, the distance between the anion and cation remains relatively constant, at ≈1 nm.^[^ ^22^ ^]^ The formation of iontronic capacitor is based on the electrical double layer (EDL) in electrochemistry. The electrical double layer (EDL) region is made up of electrons on electrodes, the Helmholtz layer, and the diffuse layer in ionic gel. Excess electron carriers on the metal electrode surface attract opposing ions in the ionic gel through electrostatic forces. The distribution of opposing ions makes up the Helmholtz layer. For the diffuse layer representing the further distribution of ions in the ionic gel, the positive and negative ions reach a Poisson–Boltzmann equilibrium state based on diffusion effects of opposing ions. The equivalent model of the EDL is two series‐connected interface capacitors. For the capacitance value of EDL, ion diffusion, interface conditions, and material properties all affect the unit area capacitance UAC (nF cm^−2^) of the EDL. The total capacitance (nF) of the EDL is the product of the unit area capacitance UAC and the contact area *A* (cm^2^) between the electrode and the ionic gel. Therefore, the capacitance of the iontronic capacitor is mainly influenced by the contact area that changes with pressure when the unit area capacitance UAC is constant.^[^ ^19^ ^]^ In designing iontronic flexible pressure sensors, attention is often focused on designing thousands of micrometer‐scale structures on a flexible ionic gel to improve the performance of iontronic flexible pressure sensors in terms of sensitivity, linearity, the limit of detection, and measurement range.^[^ ^23^ ^]^ 2020 Guo designs a graded intrafillable microstructure to increase sensitivity to 3302.9 kPa^−1^.^[^ ^24^ ^]^ 2022 Guo designed a graded interlocks microstructure to improve linearity to 0.999.^[^ ^25^ ^]^ 2022 Shen designed a full‐skin bionic double interlocked layered microstructure to improve the detection limit to 0.05 Pa.^[^ ^9^ ^]^ 2023 Cheng used laser‐induced gradients to design a programmable microstructure to broaden the range to 1800 kPa.^[^ ^26^ ^]^ Recently, the material properties of ionic gel have also been modified to improve the performance of iontronic pressure sensors.^[^ ^27^ ^]^ In addition to these factors, identifying more indicators that affect sensor performance is crucial for comprehensive evaluation, ultimately meeting specific requirements.

The majority of flexible pressure sensors are packaged in a sandwich form, comprising an upper electrode, a sensing layer, and a lower electrode that are all securely taped together.^[^ ^28^ ^]^ In the case of iontronic flexible pressure sensors, a sizable initial capacitance (several tens of pF) is produced when the electrode comes into contact with the ionic gel possessing high capacitance characteristics. To reduce the initial capacitance value, it is common practice to place a circle of flexible and elastic PDMS spacer around the ionic gel.^[^ ^29^, ^30^, ^31^ ^]^ PDMS spacer ensures that the upper and lower electrodes do not come into contact with the ionic gel simultaneously. As a result, a normal parallel‐plate capacitor rather than iontronic capacitor is generated in the initial state, leading to a very small initial capacitance of only a few pF. Nevertheless, the performance of the sensor is significantly affected by the design, which transfers some of the pressure on the flexible substrate to the PDMS spacer rather than applying it entirely to the ionic gel.

Based on the above considerations, we report a millimeter‐scale graded hollow ball arch (GHBA) structure, which is easily visible to the naked eye and has a high yield rate. GHBA structure is based on the principle that when breathing, the alveoli undergo significant contraction and relaxation, and that the arch structure in the bridge discipline can disperse vertical pressure into horizontal thrust. GHBA structure possesses the advantages of significant deformation during compression and major variations in the contact area between the electrode and the ionic gel. The fabrication of GHBA is simple and ingenious by using the idea of a double‐sided microstructure. One side employs mold casting to create convex structures, while the other side utilizes moisture (H_2_O) evaporation to form concave structures. Furthermore, a dual‐layer graded structure is designed to buffer the high‐pressure loads, thereby increasing the range of measurement. To improve sensitivity, a novel side‐by‐side package (SBSP) structure is proposed, which transfers almost all the pressure on the flexible substrate to the GHBA ionic gel film. Experimental results show that the sensor has a maximum sensitivity of 10 420.8 kPa^−1^ (R^2^ = 0.99) in a 300 kPa measurement range. It also has a fast response/recovery time of 40/35 ms and excellent repeatability, withstanding over 1800 repetitive pressure cycles of 300 kPa. The sensor is also able to detect tiny pressure as low as 0.2 Pa.

## 2 Result and Discussion

### 2.1 Design Mechanism and Fabrication Process of the Iontronic Sensor

**Figure 1:**
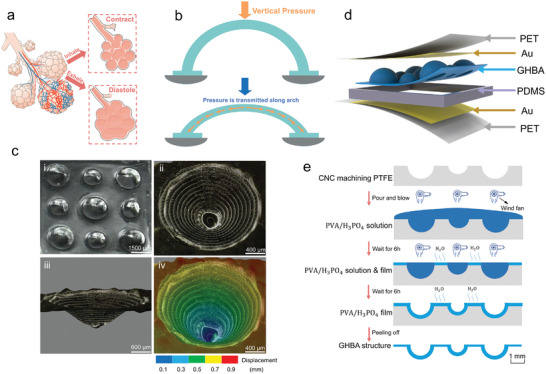
Design principles and fabrication of the GHBA structure. a) Schematic diagram of alveolar inhalation contract and exhalation diastole. b) The arch structure disperses the vertical pressure along the direction of the arch. c) 3D images of the GHBA structure on the ionic gel film: i) 3 × 3 array overall layout diagram, ii) top view of a hollow ball arch structure, iii) side upper view of a hollow ball arch structure, iv) deep measurement diagram of a hollow ball arch structure. d) Exploded view illustrating the design layout of the iontronic sensor. e) Fabrication of GHBA structure ionic gel film.

For iontronic flexible pressure sensors, the stronger the compressibility of the ionic gel film microstructure, the greater the deformation, leading to a delay in the arrival of pressure saturation.^[^ ^32^ ^]^ We extensively surveyed objects in nature with properties similar to the flexibility of ionic gel film, ultimately discovering that human pulmonary alveoli conform to and possess high compressibility.^[^ ^33^ ^]^ As shown in **Figure** **1**a, during inhalation, the alveoli contract, while during exhalation, the alveoli expand. The fact that there is plenty of air inside plays a major role. Based on the high compressibility of ionic gel film and the constant UAC, the capacitance value of an iontronic flexible pressure sensor is primarily determined by the contact area between the ionic gel film and the electrodes. As the contact area increases, the capacitance value gradually rises.

The thrust applied by the ionic gel film structure in a horizontal direction is what causes the contact area to increase. In other words, the ionic gel film structure stretches horizontally in response to a thrust in a horizontal direction, increasing the contact area. Since the pressure sensor applies the pressure only in the vertical direction, a structure that can disperse vertical pressure to horizontal thrust would be very helpful in expanding the contact area. Taking inspiration from bridge discipline, as shown in Figure 1b, an arch structure can disperse vertical pressure into horizontal thrust.^[^ ^34^ ^]^

Based on the considerations of alveoli and arch structures, a hollow ball arch shape not only ensures that the structure has a large deformation but also enables the pressure applied in the vertical direction to have significant components in all directions around the horizontal plane, thus allowing the structure to expand uniformly in the horizontal direction and extremely increasing the contact area. Currently, there is a common pursuit to create tens of thousands of micrometer‐scale structures on a 1 cm × 1 cm flexible substrate.^[^ ^35^ ^]^ Pyramids, sandpaper, spine, and leaf microstructures ranging in size from a few micrometers to hundreds of micrometers are commonly used (Table S1, Supporting Information). Since the average pressure applied to each microstructure is only 0–30 Pa, there is minimal deformation. Ensuring that each of the tens of thousands of microstructures is exactly like the ideal shape during fabrication is also a challenging task. In addition, each structure has little strength and is susceptible to accidental damage because of its small size.^[^ ^36^ ^]^ Therefore, we determined that the hollow ball arch microstructure should be millimeter scale, with a 3 × 3 matrix uniformly distributed on a 1 cm × 1 cm flexible substrate and an average pressure of 0–30 kPa applied to each structure.

At the same time, we design two levels of hollow ball arch structures. This graded design, on the one hand, it protects the first‐level hollow ball arch structures from damage under high pressure by allowing the second‐level hollow ball arch structures to share some of the pressure, thereby increasing the measurement range. On the other hand, the first‐level hollow ball arch structures become less compressible as the contact area grows under high pressure. The newly emerged second‐level hollow ball arch structures are more susceptible to compression under high pressure, thus effectively increasing the degree of contact area expansion and sensitivity.^[^ ^37^ ^]^

Based on the above considerations, we design a 3 × 3 matrix of graded hollow ball arch (GHBA) structure (Figure 1c‐i) that consists of two different kinds of hollow ball arch structures: a taller one with a diameter of 2 mm and a shorter one with a diameter of 1.5 mm. 3D images (Figure 1c‐ii,iii) and depth cloud image (Figure 1c‐iv) show the hollow ball structure in detail. The backside of the GHBA structure is shown in Figure S1 (Supporting Information). To reduce the initial capacitance value, PDMS spacers are placed around the GHBA structure to ensure that the upper and lower electrodes do not simultaneously contact GHBA. As illustrated in Figure 1d, the iontronic pressure sensor consists of the GHBA structure, gold‐plated PET flexible substrate, and PDMS spacer. The equivalent circuit diagram of the GHBA structure is shown in Figure S2 (Supporting Information).

The fabrication process of the GHBA structure is simple and ingenious. Microstructures are created on both the front and back sides of the PVA/H_3_PO_4_/H_2_O ionic gel film. On the front side, semi‐spherical convex structures are formed by reverse molding a mold. On the back side, semi‐spherical concave structures are formed by increasing the evaporation of moisture (H_2_O) from the ionic gel at the convex structure using a wind gun. Figure 1e shows the detailed fabrication process of GHBA (physical diagram displayed in Figure S3 (Supporting Information). First, polytetrafluoroethylene (PTFE) is precisely carved into a 3 × 3 semi‐spherical concave matrix with diameters of 2 and 1.5 mm using a CNC machine. Subsequently, the PVA/H_3_PO_4_/H_2_O ionic gel solution is dropped onto the PTFE mold with the semi‐spherical concave structure. During curing at room temperature, the moisture (H_2_O) in the PVA/H_3_PO_4_/H_2_O ionic gel solution gradually evaporates, ultimately forming an ionic gel film. On the PTFE mold, the thickness of the ionic gel solution at the concave structure is significantly higher than that at the flat surface, resulting in different curing times for the concave and flat areas. To expedite and increase the evaporation of moisture (H_2_O) at the concave structure, a 2 mm diameter nozzle of a wind gun is used to blow air directly above the concave structure. Surprisingly, after 6 h, the flat areas without semi‐spherical concave structures have completely solidified into thin ionic gel film, while the areas with semi‐spherical concave structures remain in a liquid state. Subsequently, the moisture (H_2_O) at the concave structures continues to evaporate, resulting in a reduction in the thickness of the ionic gel at these locations. After 12 h, both the flat and semi‐spherical concave structures of the ionic gel solution have completely solidified. Due to the hydrophobic nature of PTFE, it could be peeled off from the PTFE mold, ultimately resulting in the formation of the graded hollow ball arch (GHBA) structure.

Finally, using magnetron sputtering, 100 nm gold layer is deposited on 125 µm PET substrate to serve as the conductive and flexible upper and lower substrates for the iontronic sensor.

**Figure 2:**
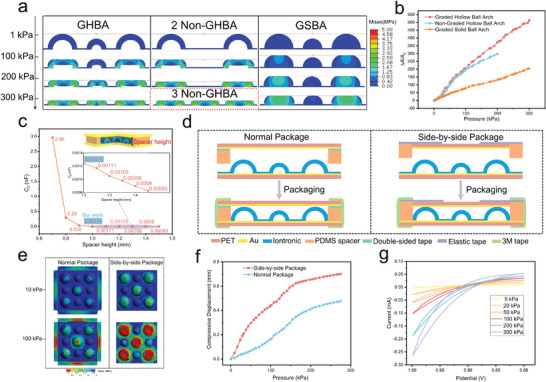
FEA results, initial capacitance design, and package structure design. a) Stress distribution of FEA simulation results for four different structures under pressures up to 300 kPa. b) Rate of change in the contact area between electrode and ionic gel film with different structures in the pressure range of 0–300 kPa. c) The initial capacitance value of GHBA structure varies with spacer height. d) Structures of the normal package and side‐by‐side package. e) FEA simulation results show the deformation and stress distribution of GHBA structure under the pressure of 10 and 100 kPa for normal package and side‐by‐side package. f) Compressive displacement of the iontronic sensor with the normal package and side‐by‐side package. g) CV curves were tested in two electrode modes at scan rate 10 mv s^−1^ with various applied pressures.

As shown in **Figure** **2**a, we simulate the graded hollow ball arch (GHBA) structure, non‐graded hollow ball arch (Non‐GHBA) structure, and graded solid ball arch (GSBA) structure using the ABAQUS finite element analysis software.^[^ ^24^ ^]^ The simulation results indicate that the compressibility of GHBA is significantly improved compared to GSBA. Furthermore, GHBA structure provides a cushioning effect for high‐pressure loads compared to Non‐GHBA with two microstructures, and its maximum tolerable pressure is essentially consistent with the Non‐GHBA structure with three microstructures. In this simulation, it can be observed that GHBA structure undergoes three main stages of change. In Step 1, the first level height structure is compressed, leading to an increase in contact area and capacitance. In Step 2, the second level height structure starts to be compressed, increasing the contact area while alleviating the pressure on the first level height structure. Compared to nongraded structures, the graded design not only improves sensitivity but also significantly increases the measurement range. From the simulation result, it can be concluded that the sensitivity of GHBA is higher than Non‐GHBA with only two first‐level height structures, while the measurement range is almost the same as that of Non‐GHBA with three first‐level height structures. In Step 3, the bottom of GHBA gradually comes into contact with the lower electrode, improving sensitivity under high pressure. Additionally, as shown in Figure 2b, we exported the contact area change rate with pressure variation from ABAQUS, which indicates that graded hollow structure significantly increases the contact area compared to graded solid structures and greatly extends the measurement range compared to non‐graded hollow structure.

### 2.2 Design of Initial Capacitance and Package Structure

The iontronic capacitor has high capacitance values even with small contact areas, while the normal parallel plate capacitor has significantly lower capacitance values in comparison. A circle of flexible and elastic PDMS spacer is placed around the GHBA structure, and a PET‐Au conductive flexible substrate is placed on top of the PDMS spacer. The PDMS spacer can determine whether the PET‐Au conductive flexible substrate comes into contact with the GHBA structure, thereby determining whether the initial capacitance is an iontronic capacitance or a normal parallel plate capacitance. The initial capacitance is an iontronic capacitance with a large value when the PDMS spacer is absent. Moreover, the restoration of the initial capacitance to its original state relies entirely on the elasticity of the GHBA structure and cannot steadily return to the minimum value (Figure S4, Supporting Information). The initial capacitance is a normal parallel plate capacitance with a small value when the PDMS spacer is present. PDMS not only reduces the initial capacitance value but also provides support to the flexible substrate, helping steadily return to the initial state after releasing pressure. For a normal parallel plate capacitor, the Graded hollow ball arch (GHBA) structure, with a height of 1 mm and compressible air inside, not only enhances sensitivity by reducing stiffness but also lowers the initial capacitance of the sensor. The thickness of the concave part of the GHBA structure film is approximately 200 µm. The initial capacitance of GHBA structure is equivalent to a 200 µm thick film in 800 µm height of air (Figure S5, Supporting Information), which is lower than a 1000 µm thick film and a 200 µm thick film. The formula *C*_0_ = ε_0_ *S*/*d* is used for calculating the initial capacitance. Due to the 1 mm height (*d*) and the relative dielectric constant ε_0_ approaching close to one,^[^ ^38^ ^]^ the initial capacitance *C*_0_ is reduced to ≈1 pF, thus favoring an increase in the relative change in capacitance (∆*C*/*C*_0_). Figure 2c shows when the spacer height is less than 1 mm, GHBA comes into contact with the PET‐Au conductive flexible substrate, resulting in an initial capacitance that is not the normal parallel plate capacitance, but rather iontronic capacitance in the range of ten to thousands of pF. When the spacer height is 1 mm, GHBA and the Au‐PET conductive flexible substrate are just out of contact, resulting in a normal parallel plate capacitance of only 0.00119 nF (Figure S6, Supporting Information). As the spacer height continues to increase, the capacitance value will continue to decrease. However, an air gap will form between the top o GHBA structure and the PET‐Au conductive flexible substrate when the height of the PDMS spacer is more than 1 mm. At low pressure (within a few kPa), it is not a highly sensitive iontronic pressure sensor but an extremely low sensitive normal flat capacitive pressure sensor with an air gap. Therefore, it is essential to minimize the air gap between the GHBA structure and the PET‐Au conductive flexible substrate, setting the height of the PDMS spacer to 1 mm and ensuring that the GHBA structure exactly does not make contact with the PET‐Au conductive substrate. PDMS spacer with a height of 1 mm ensures that the sensor maintains a high sensitivity right from the initial state (no‐load pressure).

As shown in the left image of Figure 2d, a commonly used package structure involves placing ionic gel film and PDMS spacer between the upper and lower electrodes to prevent direct contact between the upper electrode and the ionic gel film. Then, 3 m adhesive tape is used to bond the upper and lower flexible substrates with the PDMS spacer, completing the packaging process. For this package structure, a significant amount of pressure applied to the flexible substrate is lost in the PDMS spacer and is not fully transmitted to the ionic gel film,^[^ ^39^ ^]^ as can be seen in the left image of Figure 2e. As shown in the right image of Figure 2d, we propose a novel side‐by‐side package (SBSP) structure to address this issue. In this approach, the PDMS spacer is designed to be at the same level as the top surface of the PET flexible substrate. Excellent performance elastic tape is then used to connect and secure them, while 3 m adhesive tape is used to bond the upper and lower flexible substrates with the PDMS spacer, completing the packaging. As depicted in the right image of Figure 2e, the side‐by‐side package not only ensures that the upper electrode does not make contact with the ionic gel film, but also eliminates pressure loss in the PDMS spacer, allowing the pressure applied on the flexible substrate to be almost fully transmitted to the ionic gel film. As shown in Figure 2f, the GHBA structure with a side‐by‐side package has less stiffness and larger compression displacement for the same pressure.

### 2.3 Sensing Properties of the Iontronic Sensor

**Figure 3:**
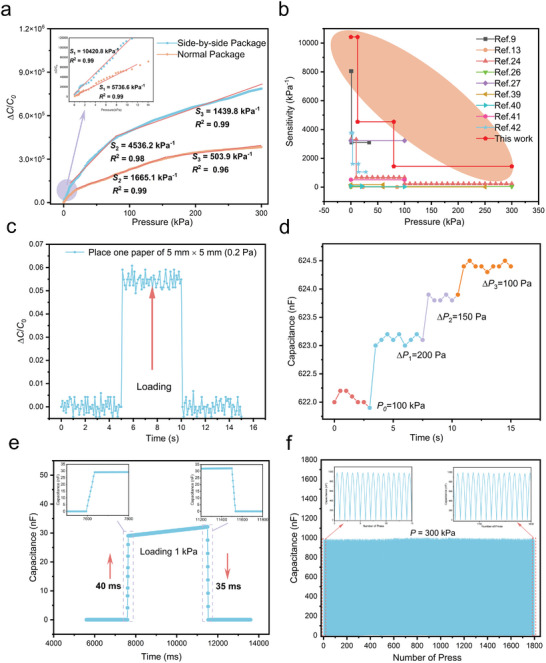
Sensing properties of the iontronic pressure sensor. a) Sensitivity and linearity with side‐by‐side package and normal package. b) Comparison of sensitivity with other iontronic pressure sensors. c) Limit of detection. d) Responses of micropressure (100−200 Pa) under pressures of 100 kPa. e) Response time and recovery time under pressure of 1 kPa. f) Mechanical durability test over 1800 cycles with 300 kPa pressure loading and releasing.

When the characteristics of the ionic gel material are determined, the unit area capacitance UAC only changes with the driving frequency (Figure S7, Supporting Information). The iontronic capacitance only changes with the contact area between the ionic gel and the PET/Au electrodes when the driving frequency is constant (1 kHz). As shown in Figure 2g, the cyclic voltammetry (CV) curve indicates an increase in the amount of electrical charge on the electrodes with the pressure and contact area becoming larger, resulting in an increase in the capacitance. The sensitivity (*S*) of iontronic flexible pressure sensor is defined as the relative change in capacitance per unit pressure, defined by the formula δ(∆*C*/*C*_0_)/δ*P*, where ∆*C* is the change in capacitance, *C*_0_ is the initial capacitance, and *P* is the applied pressure. **Figure** **3**a shows the sensitivity and linearity of the graded hollow ball arch (GHBA) structure with a side‐by‐side package (SBSP) under pressure ranging from 0 to 300 kPa. The sensitivity reached up to 10 420.8 kPa⁻¹ and linearity reached up to 0.99 in the 0–12.5 kPa range. At the same time, sensitivity remained at 4536.2 kPa⁻¹ and linearity is 0.98 in the 12.5–80 kPa range. Surprisingly, even under high pressure of 80–300 kPa, the sensitivity remained high at 1439.8 kPa⁻¹ and linearity is 0.99. Figure S8 (Supporting Information) shows the detailed variation of capacitance value with pressure. Additionally, we also test the sensitivity and linearity of the graded hollow ball arch (GHBA) structure with the normal package, which is only a third to half of the side‐by‐side package (SBSP) structure.

As shown in Figure 3b,^[^ ^9^, ^13^, ^24^, ^26^, ^27^, ^39^, ^40^, ^41^ ^]^ a comparison is made among the sensitivity of iontronic pressure sensors during the last two years. The sensitivity reported in this study exceeds that of most other articles and is notably higher than that of other sensors under high pressure (100–300 kPa). This is closely related to the deformation of the GHBA structure in the second and third stages.

The limit of detection (LOD) for pressure sensing refers to the minimum pressure that the sensor can detect. Using 10 mm × 10 mm, 10 mm × 5 mm, 5 mm × 5 mm, and 5 mm × 2.5 mm paper pieces gently placed on the sensor, it was observed that when the 5 mm × 5 mm (0.2 Pa) paper piece is placed, as shown in Figure 3c, a small change in capacitance occurred. Therefore, the pressure detection limit (LOD) for this sensor is 0.2 Pa.

It is also crucial to detect small pressure under high pressure, and this can be measured using the performance indicator of resolution. Specifically, it is defined as the ability to detect a small pressure based on a large pressure already applied. As shown in Figure 3d, with a pressure of 100 kPa already applied, we were able to clearly observe the capacitance changes when additional pressures of 200, 150, and 100 Pa were continuously applied to the device. Therefore, the minimum resolution achieved is 0.1%.

The dynamic response speed of a sensor refers to the time it takes for the sensor signal to appear or disappear when pressure is applied or released on the sensor. This can be further defined as the response time and relaxation time, respectively. The dynamic response speed is particularly important for detecting high‐frequency mechanical stimuli. On an isolation table, as shown in Figure 3e, a 1 kPa pressure is quickly applied on the surface of the sensor, and the response time is measured to be 40 ms. When the pressure is quickly released, the relaxation time is measured to be 35 ms.

The ability of a sensor to be used repeatedly over a long period of time without experiencing fatigue or signal drift is a key factor in ensuring its suitability for human or industrial use. As Figure 3f illustrates, the sensor exhibits very little drift after 1800 cycles of applying and releasing 300 kPa pressure with fatigue testing equipment.

In summary, our sensor possesses high sensitivity, a wide operating pressure range, a short response time, and high mechanical durability. These features are advantageous for sensitive, reliable, and real‐time perception of mechanical signals, laying an important foundation for applications such as human wearables and electronic skins for robots.

### 2.4 Wearable Device and Electronic Skin Applications

Due to soft and flexible characterization, flexible pressure sensors can conform to the surface of the human body, making them suitable for application in human health monitoring.^[^ ^42^, ^43^ ^]^ Dumbbell curl exercise is effective for strengthening the biceps and improving muscle mass. However, incorrect fitness positions can lead to muscle imbalance and excessive pressure on the elbow joint and wrists.^[^ ^44^ ^]^ Using iontronic flexible pressure sensors to measure pressure at key points of the body enables timely and cost‐effective correction of improper fitness positions.

**Figure 4:**
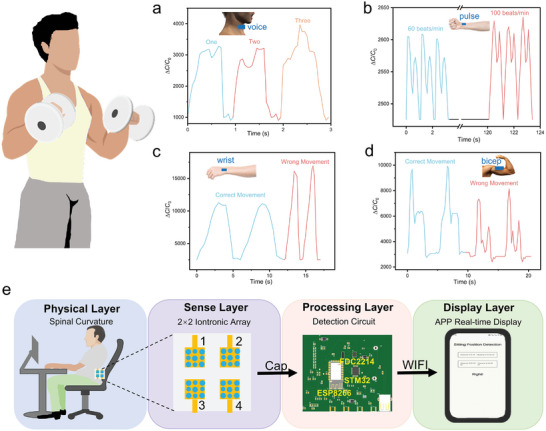
Wearable device applications. a) Capacitance changes for monitoring the pronunciation of different words. b) Capacitance changes during pulse rate at 70 and 100 beats/min. c) Capacitance changes in correct and incorrect wrist position. d) Capacitance changes at the biceps during correct and incorrect positions. e) Wireless sitting position detection system.

The number of dumbbell lifts can be recorded by the different pressure values generated through vocal cord vibrations during pronunciation. As shown in **Figure** **4**a, when speaking “ONE,” “TWO,” and “Three” at 70 decibels, a flexible pressure sensor detects distinct waveforms. Pulse rate is a vital indicator reflecting the pumping function of the heart and plays a crucial role in assessing the physiological status.^[^ ^45^, ^46^ ^]^ By using flexible pressure sensors to detect a pulse, real‐time monitoring of health during exercise is achievable. Figure 4b shows pulse signals at rates of 90 and 130 beats/min. When performing dumbbell curls, the muscles in the wrist need to provide stable support to prevent the arm from shaking or twisting while lifting the dumbbell. Figure 4c shows capacitance changes of the iontronic sensor in correct and incorrect wrist movement positions. Incorrect posture during dumbbell curls can prevent effective training of the biceps muscles. Figure 4d shows capacitance changes of the iontronic sensor when the biceps are trained correctly and incorrectly.

Figure 4e shows a 2 × 2 array of GHBA iontronic pressure sensors that can be attached to the human spine (2 × 2 matrix PET/Au displayed in Figure S9, Supporting Information). When the sitting position is incorrect and the spine bends, the capacitance values outputted by the sensor array will change. The capacitance values can be collected using the capacitance sensing chip FDC2214 and transmitted to a mobile app via WiFi using the ESP8266 chip. This allows for real‐time display of the capacitance values and assessment of the correct sitting position.

**Figure 5:**
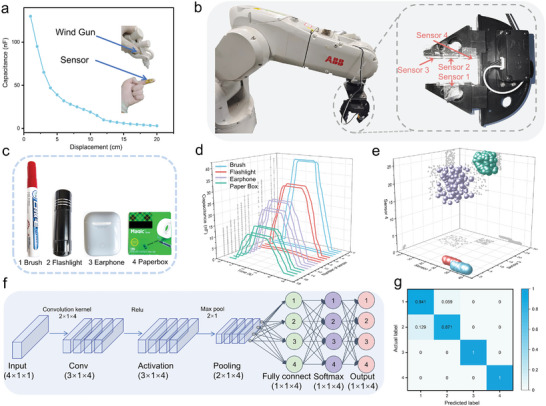
Electronic skin application. a) Capacitance changes with 80 psi wind gun position changing. b) Physical diagram of robotic arm and the location of the sensors. c) Different shapes and hardness objects: brush, flashlight, earphones, and paper box. d) Real‐time capacitance responses of four iontronic sensors on robotic arm during grasping process. e) 400 sets of collected data represented in a 4D graph. f) 1D CNN architecture diagram. g) Confusion matrix showing a recognition accuracy of 95.3%.

Flexible pressure sensors can serve as electronic skin, enabling robots to acquire both non‐contact and contact tactile perception capabilities.^[^ ^47^, ^48^, ^49^ ^]^ In terms of noncontact tactile perception, as shown in **Figure** **5**a, when the sensor is attached to a finger and an 80 psi wind gun is used to blow air, it can be observed that as the distance between the wind gun and the finger decreases, the capacitance value output by the sensor increases.

In the context of tactile perception through contact, as illustrated in Figure 5b, four sensors are attached to the mechanical gripper of a robotic arm to record the variations in capacitance output as the gripper grasps and releases objects. Because the four sensors are located in different locations, each sensor receives a different force value, which changes the capacitance outputs. Grip and release objects repetitively in different shapes and hardness such as a brush, flashlight, earphones, and paper box illustrated in Figure 5c.

When gripping a brush and a flashlight, they are in contact with the front face of sensors one and two, resulting in a significantly large capacitance value. At the same time, they are in contact with the side face of sensors three and four, resulting in a very small capacitance value. When gripping an earphone and a paper box, they are in contact with the front face of all four sensors (1, 2, 3, and 4), and the capacitance values of all four sensors are basically the same. However, since the earphones are harder, the capacitance value is larger than that of the cardboard box. Figure 5d shows real‐time capacitance responses of four iontronic sensors on the robotic arm during the grasping (brush, flashlight, earphone, and paper box) process. Figure 5e shows the results of 400 repeated gripping and releasing tests using a 4D scatter plot (the size of the sphere represents the value of sensor two). A 1D‐CNN neural network is utilized as the “brain” of the robotic arm,^[^ ^50^ ^]^ with a grid structure depicted in Figure 5f. To define the feature of an object, the input layer uses the maximum output capacitance values from the four sensors that are attached to the gripper (4 × 1 × 1). After passing through four 2 × 1 mean convolutional kernels, a convolutional layer (3 × 1 × 4) is obtained, followed by an activation layer (3 × 1 × 4) through the use of the ReLu function. Subsequently, a pooling layer (2 × 1 × 4) is obtained using a 2 × 1 window and a stride of one. The data then passes through fully connected layers and a softmax layer to classify the results into four categories. As depicted in Figure 5g, the confusion matrix of the CNN network is obtained by testing it with 100 groups of new data, and the accuracy is up to 95.3%.

## 3 Discussion

According to the significant deformation of alveoli and the ability of the arch to disperse vertical pressure into horizontal thrust, a hollow ball arch structure is proposed. Two level heights hollow ball arche structure are designed for a 3 × 3 array. The structure is cleverly fabricated using a double‐sided microstructure. The convex structure is formed by using a mold on the front side, while the concave structure is formed by evaporating moisture during the curing process on the backside. A novel side‐by‐side package (SBSP) structure is introduced to ensure that the pressures applied on the flexible substrate of the sensor are maximally transferred to the GHBA microstructure, thereby improving sensitivity. Consequently, this sensor exhibits a maximum sensitivity of 10 420.8 kPa^−1^ within a range of 0.2–300 kPa. It achieves a resolution of 0.1% under 100 kPa, with a rapid response and recovery time of 40/35 ms. It can endure 1800 compressions/releases at 300 kPa without fatigue, and it shows no signal drift. Ultimately, this sensor has been successfully applied in wearable devices for detecting dumbbell exercises and sitting positions. In the field of robotic electronic skin, it enables non‐contact detection of wind field position and object classification and prediction with the CNN model.

## 4 Experimental Section

### Preparation of GHBA Ionic Film

The ionic gel film of GHBA structure was prepared according to the following procedure. First, using a CNC machine, mill 3 × 3 hemispheres on a 1 cm × 1 cm PTFE block. Place 2 cm diameter hemispheres at the four corners and the center, and 1 cm diameter hemispheres along the edges of the PTFE block. Second, 4 g of polyvinyl alcohol (PVA, Mw ≈145 000, from Aladdin Industrial Corporation) was dissolved into 36 g of deionized water. Stir at 90 °C using a water bath for 2 h until completely dissolved, resulting in a colorless and transparent solution. Third, the PVA solution cooled to room temperature (22 °C), 3.3 mL H_3_PO_4_ (AR, ≧ 85%, Beijing J&K Scientific Co., Ltd.) was added and stirred for 2 h. Finally, The PVA/H_3_PO_4_ solution was then poured onto the PTFE graphic mold and cured under an wind gun for 12 h.

### Preparation of the Iontronic Pressure Sensor

Use Sputter Magnetron Sputtering (DISCOVERY‐635) to deposit a 100‐nanometer thick layer of gold onto the surface of flexible Polyethylene Terephthalate (PET) film (125 micrometers thick), obtaining flexible PET‐Au electrodes. Use a 3D printed 2 × 2 array mold as a mask plate and perform magnetron sputtering to obtain a 2 × 2 array of PET‐Au electrodes. In a side‐by‐side package configuration, bond the PDMS spacer to the surface of PET‐Au electrodes in a parallel arrangement using elastic tape. Place the GHBA structure ion gel film on the lower PET‐Au electrode, and finally was edge‐packaged with 3 m elastic tape.

### Finite Element Analysis

FEA was performed using the commercial package ABAQUS 2021 and ANSYS 18.0. The ABAQUS 2D planar was used to simulate the deformation behavior and contact area of the GHBA structure under 0–300 kPa. The ANSYS Workbench was used to simulate the stress contour of the GHBA structure with the normal package and side‐by‐side package. All contact interactions were assumed to be frictionless without penetration. The ionic gel PVA/H_3_PO_4_ was treated as a hyperelastic material with a nearly incompressible property by the neo‐Hookean model, and the electrode PET‐Au was modeled as a linear elastic material. The Young's modulus of PVA/H_3_PO_4_ is set at 2.5 MPa according to experimental measurement. The Young's modulus of PET‐Au is set at 4000 MPa.

### Characterization and Measurements

The graded hollow ball arch structure of the PVA/H_3_PO_4_ film was characterized by using a digital microscope (DVM6, Leica). The capacitance was measured by using an LCR meter (E4980AL, KEYSIGHT). The external pressure was applied using a computer‐controlled electronic universal testing machine (UTM2102, Shenzhen Suns Technology Stock Co., Ltd.). The CV curve was measured by using an electrochemical analysis system at a scan rate 10 mv s^−1^ (CHI660A, CH Instruments). All capacitance signals were measured under a frequency of 1 kHz and a voltage of 1 V.

## Conflict of Interest

The authors declare no conflict of interest.

## Supporting information

Supporting Information

Supplemental Video 1

Supplemental Video 2

## Data Availability

The data that support the findings of this study are available from the corresponding author upon reasonable request.

## References

[1] Y. Li , D. Yang , Z. Wu , F.‐L. Gao , X.‐Z. Gao , H.‐Y. Zhao , X. Li , Z.‐Z. Yu , Nano Energy 2023, 109, 108324.

[2] K. Meng , X. Xiao , W. Wei , G. Chen , A. Nashalian , S. Shen , X. Xiao , J. Chen , Adv. Mater. 2022, 34, 2109357. 10.1002/adma.202109357 35044014

[3] Z. Huang , B. Chen , B. Ren , D. Tu , Z. Wang , C. Wang , Y. Zheng , X. Li , D. Wang , Z. Ren , S. Qu , Z. Chen , C. Xu , Y. Fu , D. Peng , Adv. Sci. 2023, 10, 2204925. 10.1002/advs.202204925 PMC9875687 36372543

[4] Y. Shen , W. Yang , F. Hu , X. Zheng , Y. Zheng , H. Liu , H. Algadi , K. Chen , Adv. Compos. Hybrid Mater. 2022, 6, 21.

[5] Q. Wan , Q. Chen , M. A. Freithaler , S. R. Velagala , Y. Liu , A. C. To , A. Mahajan , R. Mukkamala , F. Xiong , Adv. Healthcare Mater. 2023, 12, 2202461. 10.1002/adhm.202202461 PMC11061714 36942993

[6] Y. Wang , M. L. Adam , Y. Zhao , W. Zheng , L. Gao , Z. Yin , H. Zhao , Nano‐Micro Lett. 2023, 15, 55. 10.1007/s40820-023-01013-9 PMC9936950 36800133

[7] Y. Yu , J. Li , S. A. Solomon , J. Min , J. Tu , W. Guo , C. Xu , Y. Song , W. Gao , Sci. Rob. 2022, 7, eabn0495. 10.1126/scirobotics.abn0495 PMC9302713 35648844

[8] R. Yang , W. Zhang , N. Tiwari , H. Yan , T. Li , H. Cheng , Adv. Sci. 2022, 9, 2202470. 10.1002/advs.202202470 PMC9475538 35835946

[9] H. Niu , H. Li , S. Gao , Y. Li , X. Wei , Y. Chen , W. Yue , W. Zhou , G. Shen , Adv. Mater. 2022, 34, 2202622. 10.1002/adma.202202622 35648867

[10] Q. Yang , Z. Ye , R. Wu , H. Lv , C. Li , K. Xu , G. Yang , Adv. Mater. Technol. 2023, 8, 2300561.

[11] Y. Huang , L. Zhao , M. Cai , J. Zhu , L. Wang , X. Chen , Y. Zeng , L. Zhang , J. Shi , C. F. Guo , Adv. Healthcare Mater. 2023, 12, 2301838. 10.1002/adhm.202301838 37602671

[12] S. Li , J. Huang , M. Wang , K. Deng , C. Guo , B. Li , Y. Cheng , H. Sun , H. Ye , T. Pan , Y. Chang , Adv. Sci. 2023, 10, 2370227. 10.1002/advs.202304106 PMC10667827 37737619

[13] P. Wang , J. Liu , W. Yu , G. Li , C. Meng , S. Guo , Nano Energy 2022, 103, 107768.

[14] A. Chhetry , J. Kim , H. Yoon , J. Y. Park , ACS Appl. Mater. Interfaces 2019, 11, 3438. 30585486 10.1021/acsami.8b17765

[15] Y. Wu , Y. Ma , H. Zheng , S. Ramakrishna , Mater. Des. 2021, 211, 110164.

[16] H. Xu , L. Gao , H. Zhao , H. Huang , Y. Wang , G. Chen , Y. Qin , N. Zhao , D. Xu , L. Duan , X. Li , S. Li , Z. Luo , W. Wang , Y. Lu , Microsyst. Nanoeng. 2021, 7, 92. 34804586 10.1038/s41378-021-00318-2 PMC8599697

[17] Y. Ding , T. Xu , O. Onyilagha , H. Fong , Z. Zhu , ACS Appl. Mater. Interfaces 2019, 11, 6685. 30689335 10.1021/acsami.8b20929

[18] C. Yang , D. Zhang , D. Wang , H. Luan , X. Chen , W. Yan , ACS Appl. Mater. Interfaces 2023, 15, 5811. 36648277 10.1021/acsami.2c18989

[19] Y. Chang , L. Wang , R. Li , Z. Zhang , Q. Wang , J. Yang , C. F. Guo , T. Pan , Adv. Mater. 2021, 33, 2003464. 10.1002/adma.202003464 33346388

[20] B. Nie , S. Xing , J. D. Brandt , T. Pan , Lab Chip 2012, 12, 1110. 22311169 10.1039/c2lc21168h

[21] B. Nie , R. Li , J. Cao , J. D. Brandt , T. Pan , Adv. Mater. 2015, 27, 6055. 26333011 10.1002/adma.201502556

[22] V. Amoli , J. S. Kim , S. Y. Kim , J. Koo , Y. S. Chung , H. Choi , D. H. Kim , Adv. Funct. Mater. 2020, 30, 1904532.

[23] C. Zhao , Y. Wang , G. Tang , J. Ru , Z. Zhu , B. Li , C. F. Guo , L. Li , D. Zhu , Adv. Funct. Mater. 2022, 32, 2110417.

[24] N. Bai , L. Wang , Q. Wang , J. Deng , Y. Wang , P. Lu , J. Huang , G. Li , Y. Zhang , J. Yang , K. Xie , X. Zhao , C. F. Guo , Nat. Commun. 2020, 11, 209. 31924813 10.1038/s41467-019-14054-9 PMC6954251

[25] N. Bai , L. Wang , Y. Xue , Y. Wang , X. Hou , G. Li , Y. Zhang , M. Cai , L. Zhao , F. Guan , X. Wei , C. F. Guo , ACS Nano 2022, 16, 4338. 35234457 10.1021/acsnano.1c10535

[26] R. Yang , A. Dutta , B. Li , N. Tiwari , W. Zhang , Z. Niu , Y. Gao , D. Erdely , X. Xin , T. Li , H. Cheng , Nat. Commun. 2023, 14, 2907. 37264026 10.1038/s41467-023-38274-2 PMC10235028

[27] J. Li , J. Li , Y. Tang , Z. Liu , Z. Zhang , H. Wu , B. Shen , M. Su , M. Liu , F. Li , ACS Nano 2023, 17, 5129. 36876910 10.1021/acsnano.3c00516

[28] Y. Guo , M. Zhong , Z. Fang , P. Wan , G. Yu , Nano Lett. 2019, 19, 1143. 30657695 10.1021/acs.nanolett.8b04514

[29] B. Nie , R. Li , J. D. Brandt , T. Pan , Lab Chip 2014, 14, 1107. 24480933 10.1039/c3lc50994j

[30] Q. Su , Q. Zou , Y. Li , Y. Chen , S.‐Y. Teng , J. T. Kelleher , R. Nith , P. Cheng , N. Li , W. Liu , S. Dai , Y. Liu , A. Mazursky , J. Xu , L. Jin , P. Lopes , S. Wang , Sci. Adv. 2021, 7, eabi4563,. 34818045 10.1126/sciadv.abi4563 PMC8612682

[31] L. Gao , M. Wang , W. Wang , H. Xu , Y. Wang , H. Zhao , K. Cao , D. Xu , L. Li , Nano‐Micro Lett. 2021, 13, 140. 10.1007/s40820-021-00664-w PMC8193410 34138418

[32] Q. Liu , Y. Liu , J. Shi , Z. Liu , Q. Wang , C. F. Guo , Nano‐Micro Lett. 2021, 14, 21. 10.1007/s40820-021-00770-9 PMC8660951 34882288

[33] D. Huang , T. Liu , J. Liao , S. Maharjan , X. Xie , M. Pérez , I. Anaya , S. Wang , A. Tirado Mayer , Z. Kang , W. Kong , V. L. Mainardi , C. E. Garciamendez‐Mijares , G. García Martínez , M. Moretti , W. Zhang , Z. Gu , A. M. Ghaemmaghami , Y. S. Zhang , Proc. Natl. Acad. Sci. USA 2021, 118, e2016146118. 33941687 10.1073/pnas.2016146118 PMC8126776

[34] Y. Yang , B. Lin , W. Zhang , Arch. Civil Mech. Engin. 2023, 23, 101.

[35] J. Qin , L.‐J. Yin , Y.‐N. Hao , S.‐L. Zhong , D.‐L. Zhang , K. Bi , Y.‐X. Zhang , Y. Zhao , Z.‐M. Dang , Adv. Mater. 2021, 33, 2008267. 10.1002/adma.202008267 34240474

[36] H. Wu , B. Zhang , X. Liu , Y. Liu , J. Cui , Z. Chu , Soft Matter 2023, 19, 6468. 37404181 10.1039/d3sm00538k

[37] Z. Shi , L. Meng , X. Shi , H. Li , J. Zhang , Q. Sun , X. Liu , J. Chen , S. Liu , Nano‐Micro Lett. 2022, 14, 141. 10.1007/s40820-022-00874-w PMC9256895 35789444

[38] S. Cao , T. Wang , W. Xu , H. Liu , H. Zhang , B. Hu , W. Yu , Sci. Rep. 2016, 6, 23460. 26996323 10.1038/srep23460 PMC4800676

[39] Y. Guo , F. Yin , Y. Li , G. Shen , J.‐C. Lee , Adv. Mater. 2023, 35, 2300855. 10.1002/adma.202300855 36999198

[40] J. Tang , C. Zhao , Q. Luo , Y. Chang , Z. Yang , T. Pan , NPJ Flex. Electron. 2022, 6, 54.

[41] N. Bai , Y. Xue , S. Chen , L. Shi , J. Shi , Y. Zhang , X. Hou , Y. Cheng , K. Huang , W. Wang , J. Zhang , Y. Liu , C. F. Guo , Nat. Commun. 2023, 14, 7121. 37963866 10.1038/s41467-023-42722-4 PMC10645869

[42] Y. J. Hong , H. Jeong , K. W. Cho , N. Lu , D.‐H. Kim , Adv. Funct. Mater. 2019, 29, 1808247.

[43] H.‐R. Lim , H. S. Kim , R. Qazi , Y.‐T. Kwon , J.‐W. Jeong , W.‐H. Yeo , Adv. Mater. 2020, 32, 1901924. 10.1002/adma.201901924 31282063

[44] H.‐J. Hwang , W.‐H. Chung , J.‐H. Song , J.‐K. Lim , H.‐S. Kim , J. Mech. Sci. Technol. 2016, 30, 5329.

[45] A. Schäfer , J. Vagedes , Int. J. Cardiol. 2013, 166, 15. 22809539 10.1016/j.ijcard.2012.03.119

[46] Y. Zhao , L. Liu , Z. Li , F. Wang , X. Chen , J. Liu , C. Song , J. Yao , J. Mater. Chem. C 2021, 9, 12605.

[47] F. Zhang , Y. Zang , D. Huang , C.‐A. Di , D. Zhu , Nat. Commun. 2015, 6, 8356. 26387591 10.1038/ncomms9356 PMC4595753

[48] J. Jia , G. Huang , J. Deng , K. Pan , Nanoscale 2019, 11, 4258. 30565627 10.1039/c8nr08503j

[49] Y. H. Jung , J.‐Y. Yoo , A. Vázquez‐Guardado , J.‐H. Kim , J.‐T. Kim , H. Luan , M. Park , J. Lim , H.‐S. Shin , C.‐J. Su , R. Schloen , J. Trueb , R. Avila , J.‐K. Chang , D. S. Yang , Y. Park , H. Ryu , H.‐J. Yoon , G. Lee , H. Jeong , J. U. Kim , A. Akhtar , J. Cornman , T.‐i. Kim , Y. Huang , J. A. Rogers , Nat. Electron. 2022, 5, 374.

[50] J. Shi , Y. Dai , Y. Cheng , S. Xie , G. Li , Y. Liu , J. Wang , R. Zhang , N. Bai , M. Cai , Y. Zhang , Y. Zhan , Z. Zhang , C. Yu , C. F. Guo , Sci. Adv. 2023, 9, eadf8831. 36867698 10.1126/sciadv.adf8831 PMC9984179

