# Supplementary material for: Highly Sensitive Iontronic Pressure Sensor with Side‐by‐Side Package Based on Alveoli and Arch Structure
Source: Adv Sci (Weinh). 2024 Mar 15;11(24):2309407. doi: 10.1002/advs.202309407 (PMC11199976; doi:10.1002/advs.202309407)
Supplement: Supplementary file 1 — Supporting Information [file ADVS-11-2309407-s002.pdf]

ADVANCED SCIENCE
Open Access

Supporting Information

for *Adv. Sci.*, DOI 10.1002/advs.202309407

Highly Sensitive Iontronic Pressure Sensor with Side-by-Side Package Based on Alveoli and Arch Structure

*Zhi Ding, Weijian Li, Weidong Wang, Zhengqian Zhao, Ye Zhu, Baoyin Hou, Lijie Zhu, Ming Chen and Lufeng Che**

Supporting Information

Highly Sensitive Iontronic Pressure Sensor with Side-by-side Package Based on Alveoli and Arch Structure

Zhi Ding, Weijian Li, Weidong Wang, Zhengqian Zhao, Ye Zhu, Baoyin Hou, Lijie Zhu, Ming Chen, Lufeng Che*

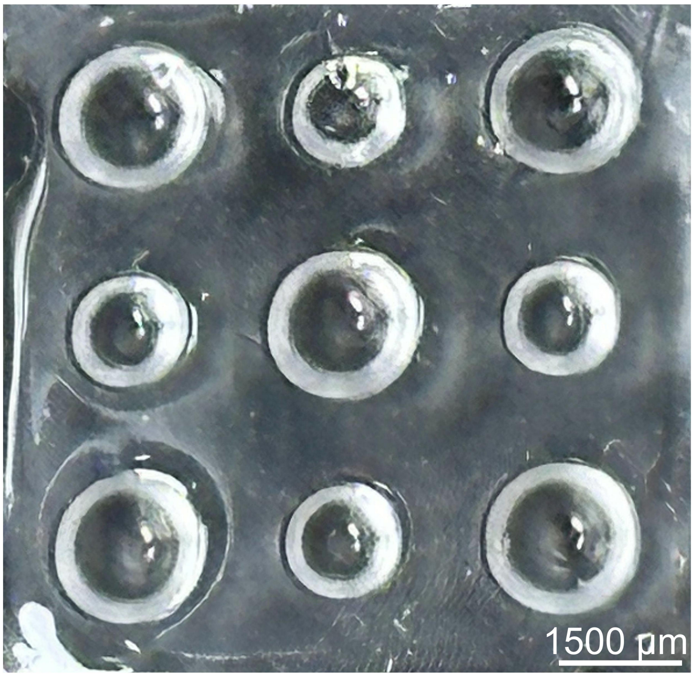

Fig. S1 The backside of the GHBA structure

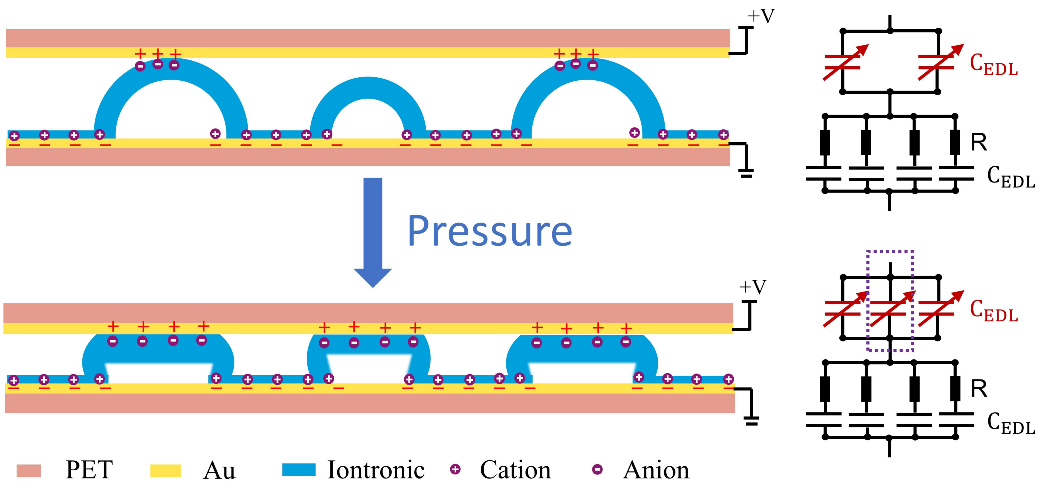

Fig. S2 Equivalent circuit diagram of GHBA structure.

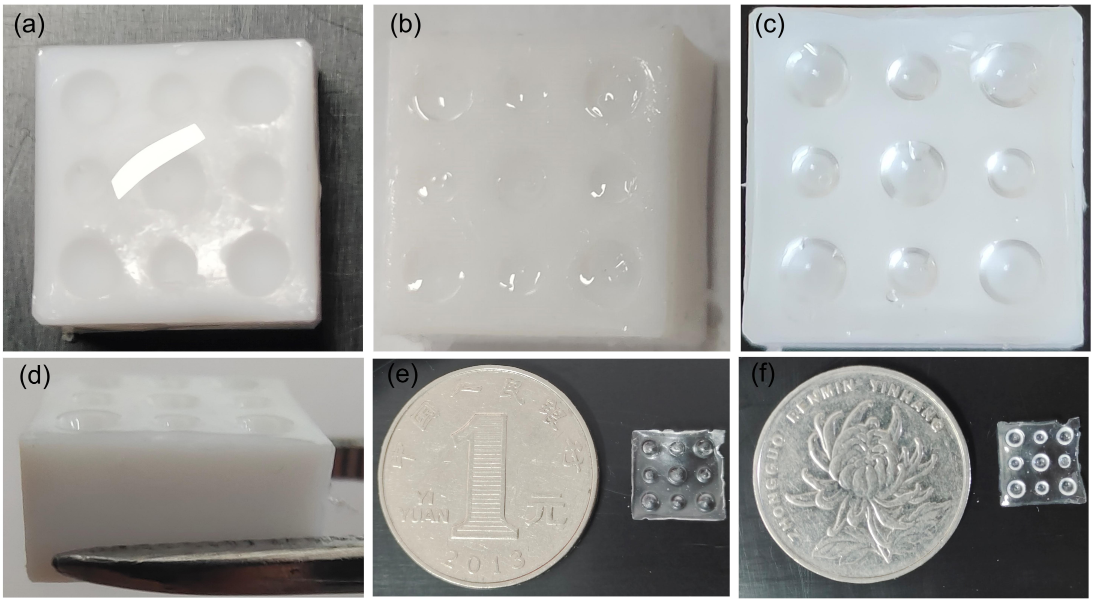

Fig. S3 GHBA structure curing process. (a) ionic gel solution state; (b) after 6 hours, ionic gel solution and film state; (c) after 12 hours, top view of ionic gel film; (d) after 12 hours, side view of ionogel film; (e) front of GHBA structure; (f) back of GHBA structure.

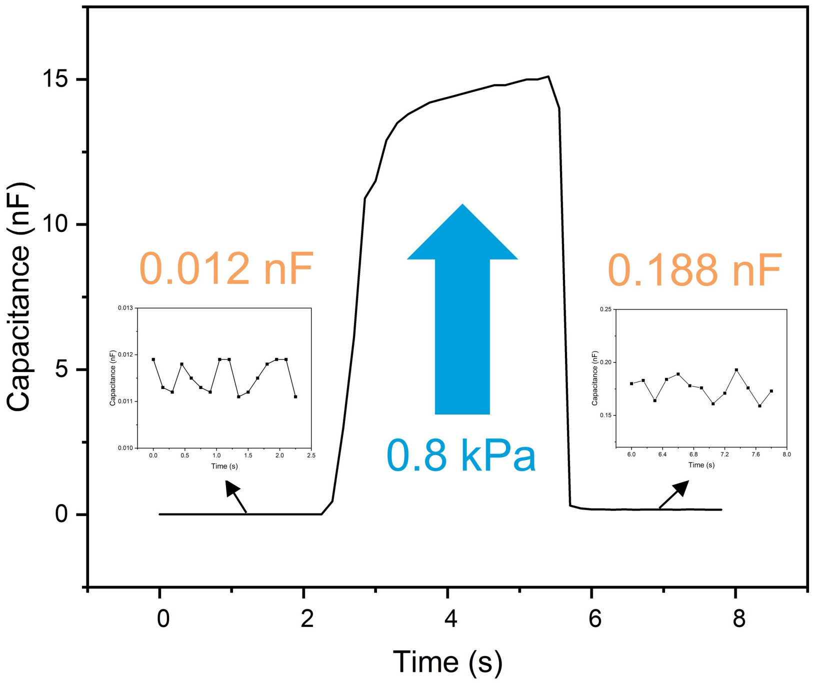

Fig. S4 Unstable recovery to the capacitance of initial state when loaded to a 0.8 kPa pressure without PDMS spacer.

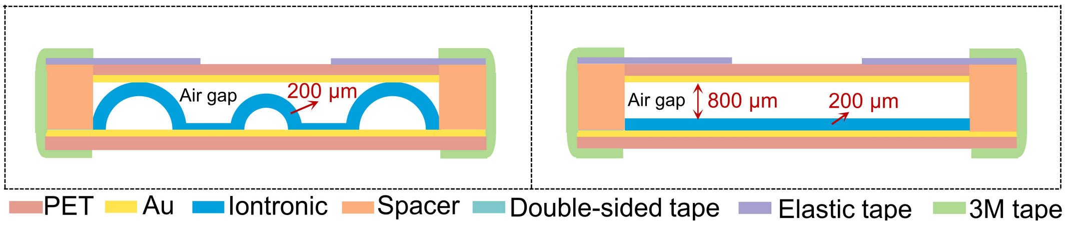

Fig. S5 Same initial capacitance for GHBA structure and 200 μm thick film with 800 μm height of air structure.

(a)

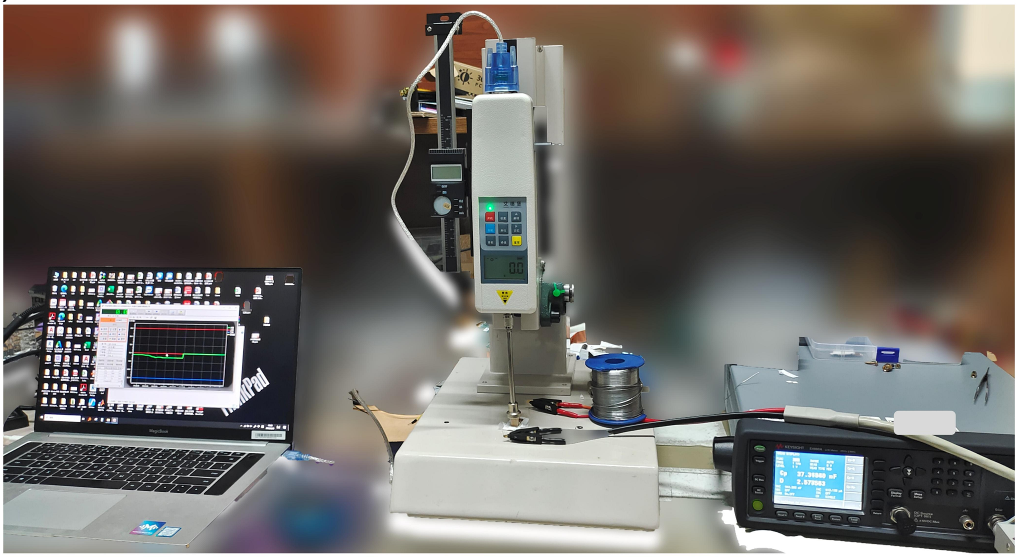

(b)

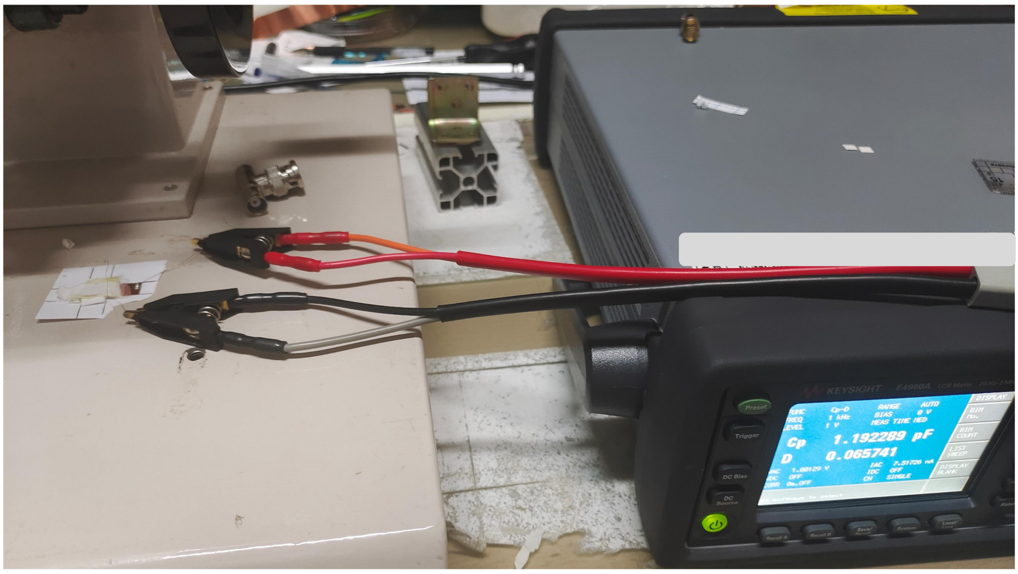

Fig. S6 Applying pressure and testing capacitive platforms. (a) computer-controlled electronic universal testing machine and LCR meter; (b) test of initial capacitance.

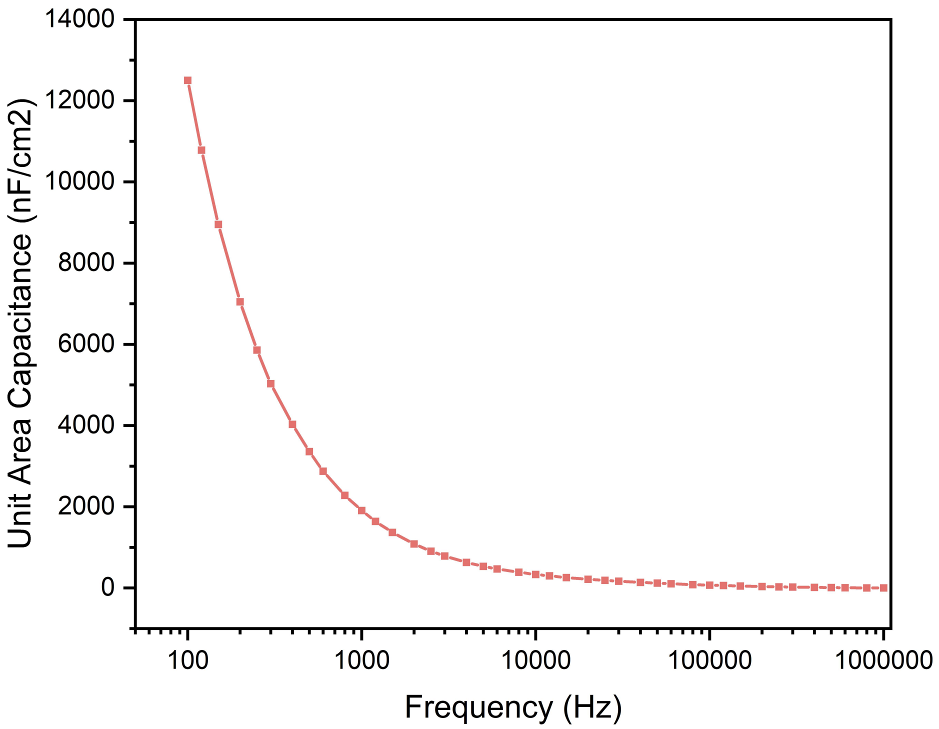

Fig. S7 Unit area capacitance of PVA/H_3PO_4 at different driving frequencies.

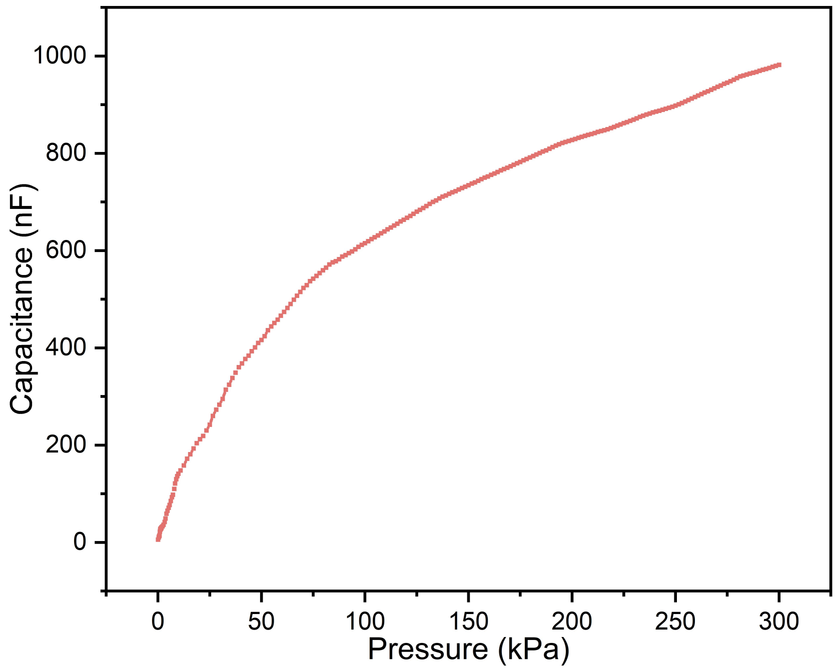

Fig. S8 The capacitance-to-pressure response of the sensor.

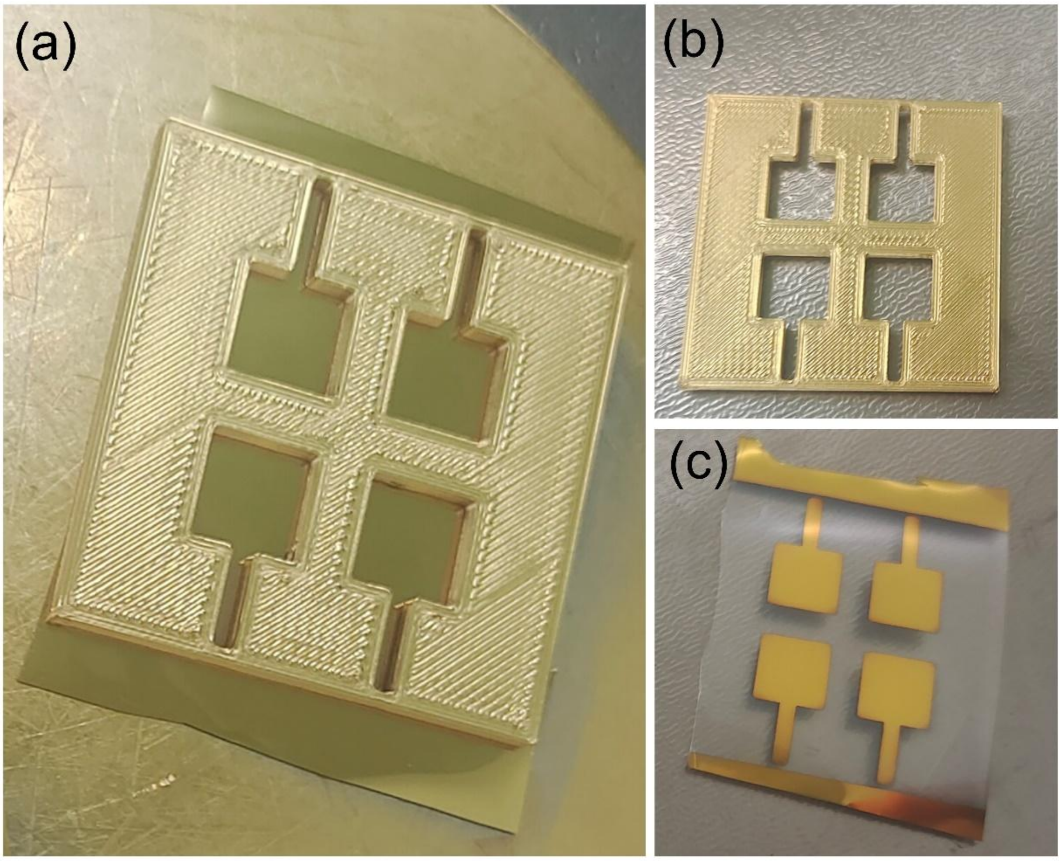

Fig. S9 Fabrication of 2 × 2 matrix PET/Au. (a) PET substrate is masked using a 3D printed mold and then magnetron sputtered with Au; (b) 3D printed mold; (c) 2×2 matrix PET/Au.

Table. S1 Microstructure shapes and dimensions for different flexible pressure sensors

Principle	Microstructure	Size of microstructure	Maximum Sensitivity	Ref.
iontronic	pyramid	5 μm	41.64 kPa^{-1}	[1]
iontronic	calathea zebrine leaf	20 μm	54.31 kPa^{-1}	[2]
iontronic	sandpaper	200 μm	131.5 kPa^{-1}	[3]
iontronic	micropillared	10 μm	33.16 kPa^{-1}	[4]
iontronic	sandpaper	2 μm	3302.9 kPa^{-1}	[5]
iontronic	ionic liquid	40 μm	83.9 kPa^{-1}	[6]
iontronic	microcone	100 μm	8053.1 kPa^{-1}	[7]
iontronic	spinousum	5 μm	2593.3 kPa^{-1}	[8]
iontronic	pyramid	500 μm	33.7 kPa^{-1}	[9]
piezoresistant	spinosum	30 μm	509.8 kPa^{-1}	[10]
piezoelectric	pyramid	10 μm	19 kPa^{-1}	[11]
capacitive	lotus leaf	7 μm	0.784 kPa^{-1}	[12]
iontronic	GHBA	1500 μm & 2000 μm	10420.8 kPa^{-1}	Our work

Supplementary References:

[1] S. H. Cho, S. W. Lee, S. Yu, H. Kim, S. Chang, D. Kang, I. Hwang, H. S. Kang, B. Jeong, E. H. Kim, S. M. Cho, K. L. Kim, H. Lee, W. Shim, C. Park, ACS Applied Materials & Interfaces **2017**, 9, 10128.

[2] Z. Qiu, Y. Wan, W. Zhou, J. Yang, J. Yang, J. Huang, J. Zhang, Q. Liu, S. Huang, N. Bai, Z. Wu, W. Hong, H. Wang, C. F. Guo, Advanced Functional Materials **2018**, 28, 1802343.

[3] A. Chhetry, J. Kim, H. Yoon, J. Y. Park, ACS Applied Materials & Interfaces **2019**, 11, 3438.

[4] P. Lu, L. Wang, P. Zhu, J. Huang, Y. Wang, N. Bai, Y. Wang, G. Li, J. Yang, K. Xie, J. Zhang, B. Yu, Y. Dai, C. F. Guo, Science Bulletin **2021**, 66, 1091.

[5] N. Bai, L. Wang, Q. Wang, J. Deng, Y. Wang, P. Lu, J. Huang, G. Li, Y. Zhang, J. Yang, K. Xie, X. Zhao, C. F. Guo, Nature Communications **2020**, 11, 209.

[6] J. Tang, C. Zhao, Q. Luo, Y. Chang, Z. Yang, T. Pan, npj Flexible Electronics **2022**, 6, 54.

[7] H. Niu, H. Li, S. Gao, Y. Li, X. Wei, Y. Chen, W. Yue, W. Zhou, G. Shen, Advanced Materials **2022**, 34, 2202622.

[8] Z. Chen, Y. Zhang, B. Zhu, Y. Wu, X. Du, L. Lin, D. Wu, ACS Applied Materials & Interfaces **2022**, 14, 19672.

[9] R. Yang, A. Dutta, B. Li, N. Tiwari, W. Zhang, Z. Niu, Y. Gao, D. Erdely, X. Xin, T. Li, H. Cheng, Nature Communications **2023**, 14, 2907.

[10] M. Yang, Y. Cheng, Y. Yue, Y. Chen, H. Gao, L. Li, B. Cai, W. Liu, Z. Wang, H. Guo, N. Liu, Y. Gao, Advanced Science **2022**, 9, 2200507.

[11] J.-H. Zhang, Z. Li, J. Xu, J. Li, K. Yan, W. Cheng, M. Xin, T. Zhu, J. Du, S. Chen, X. An, Z. Zhou, L. Cheng, S. Ying, J. Zhang, X. Gao, Q. Zhang, X. Jia, Y. Shi, L. Pan, Nature Communications **2022**, 13, 5839.

[12] M. Wang, Z. Lin, S. Ma, Y. Yu, B. Chen, Y. Liang, L. Ren, Advanced Healthcare Materials **2023**, 12, 2301005.
